# Precision cardiovascular medicine: global imperative for equity through innovation

**DOI:** 10.3389/fcvm.2026.1835607

**Published:** 2026-07-13

**Authors:** Jose R. Medina-Inojosa, Fatima Rodriguez, Francisco Lopez-Jimenez, Laurence S. Sperling

**Affiliations:** 1 Division of Cardiology, Emory Clinical Cardiovascular Research Institute, Atlanta, GA, United States; 2 Department of Medicine and Rollins School of Public Health, Emory University School of Medicine, Atlanta, GA, United States; 3 Department of Epidemiology, Emory University School of Medicine, Atlanta, GA, United States; 4 Division of Preventive Cardiology, Department of Cardiovascular Medicine, Mayo Clinic College of Medicine, Rochester, MN, United States; 5 Division of Cardiovascular Medicine, The Cardiovascular Institute, Stanford, CA, United States; 6 Center for Digital Health, Stanford University School of Medicine, Stanford, CA, United States

**Keywords:** artificial intelligence, cardiovascular disease, digital health, health equity, implementation science, low- and middle-income countries, precision medicine, World Heart Federation

## Abstract

Cardiovascular disease remains the world's leading cause of death, with a disproportionate burden falling on low- and middle-income countries. Emerging precision medicine approaches, such as artificial intelligence, multiomics, and digital diagnostics, offer transformational potential for prevention and management. Yet, without equity-centered policies, these innovations risk amplifying disparities. The World Heart Federation (WHF) proposes a framework premised on innovation as a public good and equity as a universal right. Through the World Heart Observatory, Digital Health Roadmap, and Emerging Leaders Program, the WHF operationalizes equity across governance, workforce, and care delivery. Drawing on exemplars from Africa, Latin America, and South/Southeast Asia, we outline implementation strategies and policy levers to translate science into population-level impact. Precision cardiovascular medicine can become the standard for all—but only when designed with equity from inception.

“Science is teamwork. It's about building bridges between people and ideas to make a real difference.”—Salim Yusuf, Past President, World Heart Federation

## Precision cardiovascular medicine: potential transformational opportunity for global cardiovascular health

Cardiovascular disease (CVD) remains the world's leading cause of death, with the greatest impact felt in low- and middle-income countries (LMICs) (1). In this issue of *Frontiers in Science*, Aikawa et al. (2) outlined a transformational moment for cardiovascular science, where precision medicine embraces the heterogeneity of CVD through systems biology, deployment of artificial intelligence (AI) as a translational engine linking signals to diagnostics and therapeutic discovery, and harnessing multiomics to compress healthcare development timelines and costs. The authors emphasize a crucial accelerant, the COVID-19 pandemic, which forced the rapid adoption of digital innovations, remote monitoring, and collaborative science. This serves as evidence that disruption, when matched by an urgent coordinated effort, can fast-track system redesign. However, scientific gains do not automatically translate into population benefits—especially when infectious disease and chronic care programs operate in silos rather than through an integrated infrastructure (3). Importantly, precision CVD medicine should build upon science with the goal of streamlining early detection, treatment, and longitudinal follow-up. Yet, many discovery datasets, validation cohorts, and reimbursement pathways still reflect high-income country contexts. Without a deliberate, equity-first policy, precision medicine approaches and innovations may further amplify disparities rather than reducing them. A practical framework is needed to ensure that precision cardiovascular medicine reaches everyone, everywhere, and benefits all, particularly historically underserved populations.

## World Heart Federation equity-centered initiatives for cardiovascular innovation

The World Heart Federation (WHF) proposes a simple premise: innovation is a public good and health equity is a universal right. World Heart Vision 2030 anchors this view, pairing a commitment to equity with a strategy to leverage innovation for cardiovascular health (4). The WHF Digital Health Roadmap operationalizes this vision through an eight-domain template spanning from governance to patient engagement. The message is direct: deploying precision medicine requires system-level changes in policy, workforce, data, and workflow—not just new tools (5).

As summarized in Table 1, WHF's platforms translate policy into action: the World Heart Observatory strengthens data equity (especially in LMICs), WHF Roadmaps translate evidence into context-ready guidance, and the Emerging Leaders Program builds capacity in implementation science and digital health. Across these efforts, WHF emphasizes that equity requires representation in discovery, not only validation. Moreover, cohorts that include women, rural communities, and LMIC populations are essential to fueling truly global innovation.

**Table 1:** World Heart Federation equity-centered digital innovation (2020–2025): policy levers, platforms, enablers, and exemplars.

Domain	Lever	Equity and precision mechanism	Implementation signal
Policy	WHO Geneva Charter for Well-being (2021)	Universal, inclusive systems; equity by design (context enabler)	Provides normative backing for equity language used by WHF
Policy	World Heart Vision 2030 Domain 3 (2022)	Frames precision + AI as a public good; mandates digital inclusion	Targets: −30% CVD mortality by 2030; regulatory adaptation + trial inclusion via digital data
Policy	WHF Digital Health Roadmap (2022)	Practical barriers and solutions for AI/remote/decision support	National eHealth rules, reimbursement, literacy, interoperability template
Platform	World Heart Observatory (2022)	Data equity: closes LMIC visibility gaps; supports precision metrics	One-stop CV data hub with LMIC emphasis; partner data pipelines
Platform	WHF Emerging Leaders (2024–2025)	LMIC capacity in AI + implementation science	2025 theme on AI in CVD; multi-region cohorts of implementers
Enabler	World Bank CVD Prevention Facility (2021–2026)	Financing + hardware for PoC diagnostics in primary care	18,000 PoC lipid devices (Bangladesh, Ethiopia) enable digital workflows
Enabler	CHAI market shaping (ongoing)	Affordability: lowers costs of CV meds/diagnostics for scale	Price reductions across 80 + countries in adoption curves
Enabler	African Cardiovascular Imaging Academy (2020–2023)	Workforce upskilling for echo/AI workflows	1,400 clinicians trained: ∼40% diagnostic capacity increase
Exemplar	Nigeria—AI-ECG for PPCM (6)	Task-shifting AI screening in maternal care → earlier detection	∼2x detection vs. usual obstetric care (external validation)
Exemplar	Uganda—AI-guided echo for RHD (7)	Non-expert guided acquisition with hub-and-spoke QA	Feasible identification of rheumatic MV disease by non-experts
Exemplar	Cameroon—Cardio Pad mobile ECG (8)	Tele-cardiology: remote reads expand specialist reach	Scalable wireless ECG interpreted by urban cardiologists
Exemplar	Latin America—WHO HEARTS (9)	Standardized HTN care with digital decision support	Regional primary-care uptake; protocolized control pathways
Exemplar	India—Ayushman Bharat Digital Mission (ABDM) (10)	National digital health records + telemedicine backbone enabling precision workflows at population scale	Population-scale digital infrastructure

WHF, World Heart Federation; WHO, World Health Organization; AI, artificial intelligence; CVD, cardiovascular disease; eHealth, electronic health; LMICs, low- and middle-income countries; CV, cardiovascular; PoC, point-of-care; CHAI, Clinton Health Access Initiative; echo, echocardiography; AI-ECG, artificial intelligence–enabled electrocardiography; PPCM, peripartum cardiomyopathy; RHD, rheumatic heart disease; QA, quality assurance; MV, mitral valve; ECG, electrocardiogram; WHO HEARTS, WHO HEARTS technical package for CVD management in primary healthcare; HTN, hypertension.

## The precision medicine paradox: promise vs. access

Because CVD is heterogeneous in its biological, social, and clinical determinants and trajectory, precision approaches are most valuable when tailored to local context and patient realities. As Aikawa et al. (2) emphasized, individual genetic variants, environmental exposures, and cellular responses create distinct disease patterns that demand targeted interventions (2). Yet today, access to these precision tools remains out of reach for many. Infrastructure limitations, including unreliable electricity, constrained internet connectivity, and limited point-of-care and laboratory capacity, impede deployment in many regions. Regulatory fragmentation and hurdles and delayed or absent reimbursement impede adoption, even for validated tools. Discovery inputs remain skewed toward high-income settings, limiting generalizability and risking poorer performance in settings with the highest burden of disease. Likewise, gaps in workforce capacity, ranging from digital literacy to bioinformatics expertise, limit the consistent, actionable use of precision outputs in routine care. These barriers must also be understood within the broader landscape of unmet foundational needs. In many LMIC settings, communities still lack safe drinking water, sanitation, reliable electricity, essential medications, and a functioning primary-care workforce, priorities that often precede, and compete with, investments in advanced diagnostics (2). Acknowledging this reality is critical: equity-centered precision CVD will be credible only when sequenced with, and additive to, ongoing efforts to address social determinants of health and core health-system capacity (11).

There is, however, a clear path forward that aligns with “precision public health,” which uses more accurate measurement and targeted programs to deliver the right intervention to the right person at the right time (12). Collaborative data science and globally representative biobanks can correct discovery bias and accelerate inclusive breakthroughs. AI is pivotal in linking clinical heterogeneity to actionable pathways, helping prioritize patients and subgroups most likely to benefit, especially in complex CVD phenotypes. Generative AI may shorten the usual lag between implementation and real gains in quality and productivity by lowering the skill barrier, enabling rapid iteration with clinician feedback, and leveraging increasingly available health record integration. As a facilitator, generative AI could accelerate both discovery and equitable implementation—by supporting local-language interaction, synthesizing multimodal data at the point of care, and easing specialist bottlenecks so frontline teams can implement precision outputs across all healthcare settings (13).

Importantly, innovation is already succeeding in resource-constrained settings when equity is embedded from the start. As summarized in Table 1, programs across Africa, Latin America, and South/Southeast Asia translate policy into practice through local codevelopment, integration into frontline workflows with task-shifting, and financing models that support scale. Together, they demonstrate that precision medicine can be feasible, scalable, and impactful when designed for equity from inception.

## From innovation to implementation: a practical community-centric framework

Implementing precision medicine alongside core CVD care requires an implementation-science approach focused on selecting and adapting evidence-based practices (EBPs), diagnosing local barriers, choosing fit-for-purpose implementation strategies, and evaluating adoption and sustainability. Precision tools should strengthen, not replace, EBPs. Operationalizing this agenda is non-trivial. Concrete headwinds include fragmented health information infrastructure, intermittent connectivity and power, regulatory heterogeneity, workforce shortages, affordability and supply chain constraints, and the opportunity cost of diverting scarce health system capacity from competing priorities. A pragmatic, sequenced approach that embeds precision CVD into existing primary care and non-communicable disease platforms is essential (14). A foundational framework requires six implementation strategies:

- AI and multiomics integration for diagnostics and therapeutics: Link phenotypes to treatments. Use multiomics, advanced imaging, and AI to identify subgroups, inform pharmacogenomics, and support phenotype-guided therapeutics.
- Codevelopment with local clinicians, researchers, and communities: Ensure that codesign aligns with local realities, builds trust, and gives users ownership.
- Culturally tailored design and delivery: Translate outputs into pathways that are matched to languages, literacy levels, norms, access points, and preferences.
- Interoperable, privacy-respecting digital ecosystems: Secure, standards-based data exchange enables mobile diagnostics, remote reads, and longitudinal care, even with intermittent connectivity.
- Training, task-shifting, and sustained technical support: Equip nurses, community health workers, and primary care teams to run protocols and pair them with ongoing help-desk support.
- Ongoing reassessment and adaptive evaluation: Embed iterative cycles of monitoring and user feedback to ensure that rapidly evolving technologies remain usable, effective, and responsive to evolving needs.

Delivering on these strategies requires partnerships across academia, industry, government, and civil society, with procurement rewarding value and equity and regulation enabling safe, iterative evaluation. Progress should be tracked on adoption, fidelity, feasibility, and sustainability (15, 16).

## Policy imperatives for equitable precision medicine

To move from scientific potential to real-world population impact, six levers are essential: a global fund for precision CVD to support real-world validation and delivery of pilots in LMICs; regional centers of excellence to enable local calibration, training, and capacity building; open-access toolkits for ministries of health with protocols for diagnostics and therapeutics; technical guidance and harmonization led by the WHF/WHO and regulators to align standards for safe, effective integration; streamlined equity impact assessments required before broad adoption; and sustainable financing models that blend donor, domestic, and multilateral funds, aligned with healthcare coverage and outcome-based contracts that build local capacity. Together, these levers can accelerate learning, reduce duplication, and steer resources toward solutions that are safe, effective, affordable, and equitable. We recognize that instruments such as a global fund for precision CVD will require substantial governance design, coordinated pooled financing, and deliberate alignment with existing multilateral architectures (e.g., Global Fund, Gavi vaccine alliance, WHO HEARTS, and regional non-communicable disease initiatives) to avoid parallel structures and limit administrative burden on national ministries. Operationalization should therefore be staged, beginning with regulatory harmonization, shared data standards, and anchor pilots in willing countries before broader scale-up (17).

## A call to action: accelerating innovation through inclusion

Precision cardiovascular medicine offers a powerful opportunity to deliver better care, faster, to more people. Realizing that promise requires accelerating and democratizing innovation—from AI-guided diagnostics to phenotype-guided therapies (2)—so that tools are locally developed, ethically governed, and globally shared. To correct chronic underinvestment in non-communicable diseases (18), especially CVD, this effort must fund integrated care for multiple long-term conditions and invest in both precision medicine and precision public (population) health (12). This transformation must be participatory and culturally responsive, with people and communities shaping priorities. We call on the global medical and scientific community to act boldly by scaling equitable innovation, building capacity through collaboration, and embedding equity in standards, financing, procurement, and evaluation. Critically, precision cardiovascular medicine must be pursued as a complement to, not a substitute for, sustained investment in primary healthcare, the social determinants of health, and essential public health services; its ultimate impact will be proportional to the strength of the foundations on which it is built. *The moment is now:* With partnership and vision, precision cardiovascular medicine can become the new standard for everyone, everywhere.
